# A colonic lipoma presenting as intussusception in an elderly patient

**DOI:** 10.1016/j.radcr.2025.08.030

**Published:** 2025-09-08

**Authors:** Nabil Lahlou, Nizar El Bouardi, Zakia Ettylemsany, Hajar Ouazzani, Ismail Chaouche, Amal Akammar, Meriem Haloua, Badreeddine Alami, Moulay Youssef Alaoui Lamrani, Meryem Boubbou, Mustapha Maaroufi

**Affiliations:** a Department of Adult Radiology, CHU Hassan II Fez, Sidi Mohammed Ben Abdellah University, Fez, Morocco; b Department of Mother and Child Radiology, CHU Hassan II Fez, Sidi Mohammed Ben Abdellah University, Fez, Morocco

**Keywords:** Colonic lipoma, Adult intussusception, Colocolic intussusception, Bowel obstruction, Lipoma-induced intussusception

## Abstract

Colonic lipomas are uncommon benign tumors that are usually asymptomatic but may occasionally lead to complications such as intussusception. In adults, colocolic intussusception is rare and typically associated with a structural lead point, often malignant. We report the case of a 74-year-old woman who presented with acute bowel obstruction and was found to have colocolic intussusception secondary to a colonic lipoma. Diagnosis was established through imaging, and surgical resection was performed without prior reduction. The patient recovered uneventfully. This case highlights the diagnostic challenges posed by adult intussusception and emphasizes the key role of CT imaging in identifying the underlying cause and guiding appropriate management. Early recognition and timely surgical intervention are crucial for favorable outcomes.

## Introduction

Colonic lipomas are benign, slow-growing tumors made of adipose tissue that typically occur in the submucosal layer of the colon [1]. Although they are often asymptomatic [2], in rare cases, they can serve as a lead point for bowel intussusception, a condition in which one segment of the intestine telescopes into an adjacent one, causing obstruction [3]. This report presents a case of colonic lipoma leading to colocolic intussusception, highlighting the diagnostic challenges and the importance of imaging in the management of such cases [4].

## Case report

A 74-year-old female patient with no significant medical history was admitted for management of an obstructive syndrome characterized by a 6-day history of abdominal pain, vomiting, and cessation of bowel movements. Upon clinical examination, the patient was conscious, stable, and apyretic, with no signs of abdominal distension. Her abdomen was soft to palpation, with no palpable masses or tenderness.

**Fig. 1:**
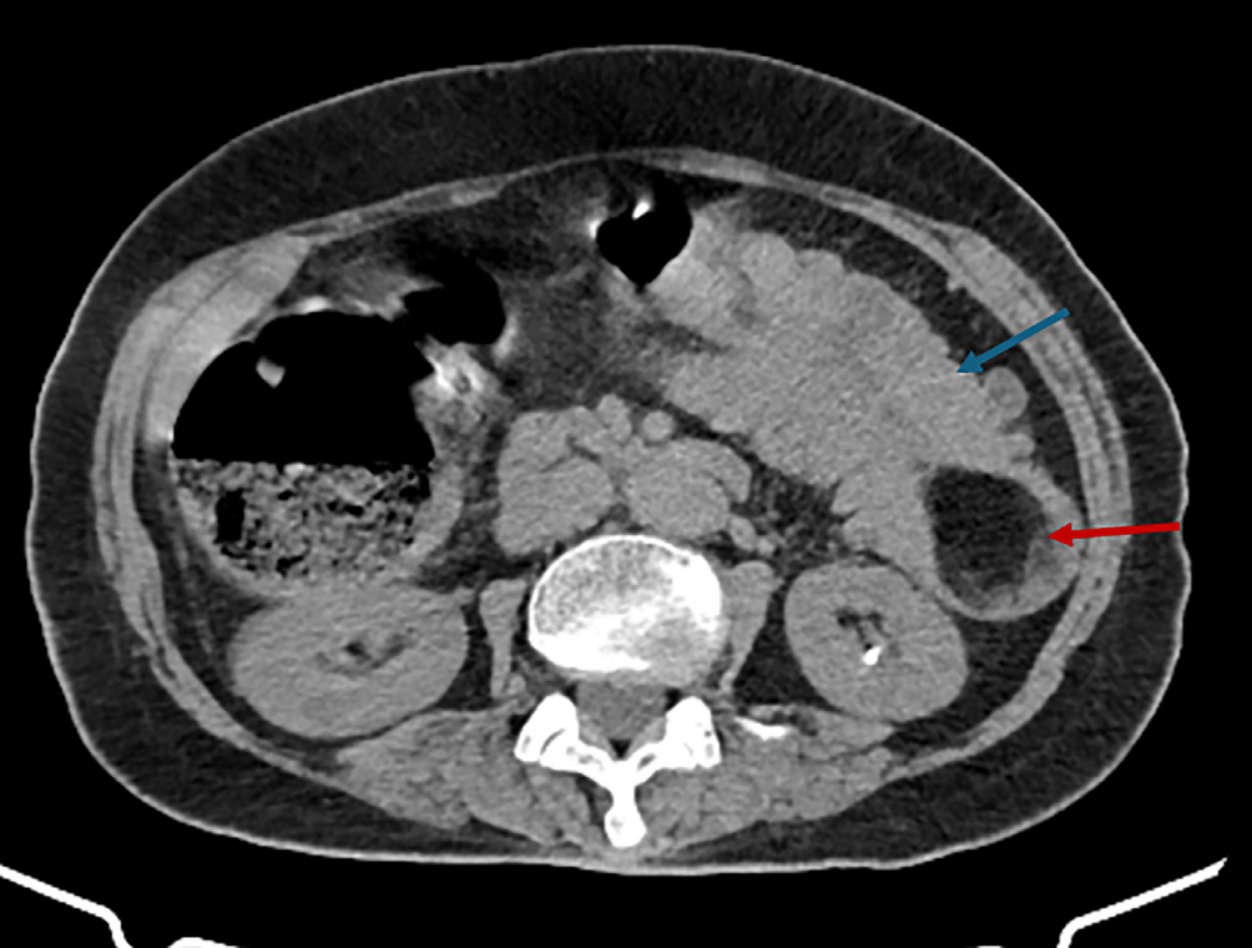
Axial nonenhanced CT showing segmental colonic wall thickening in the left abdomen (blue arrow), with an intraluminal fat-density lesion consistent with a submucosal lipoma (red arrow).

**Fig. 2:**
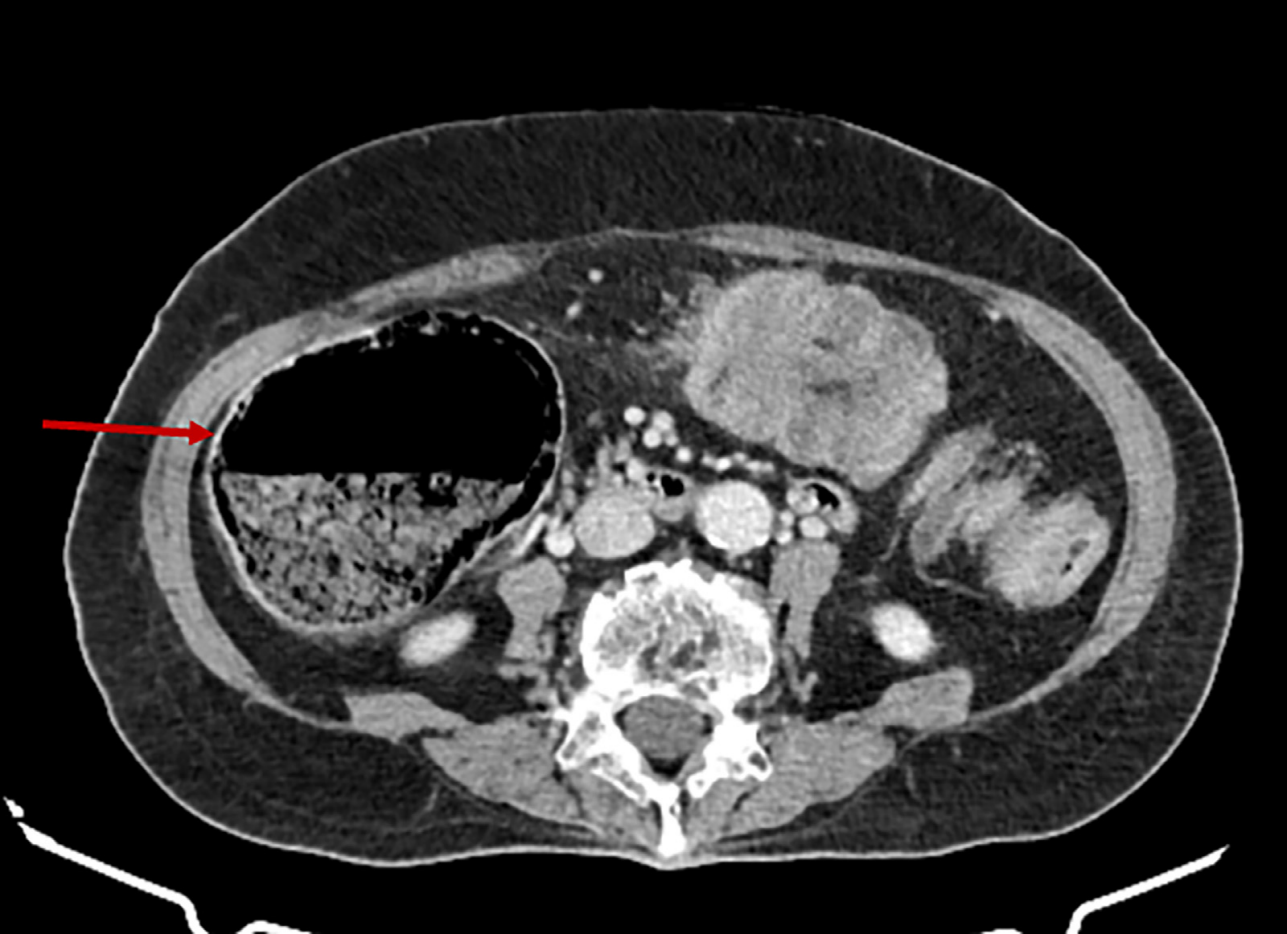
Axial contrast-enhanced CT showing marked cecal distension (red arrow), indicative of upstream colonic obstruction related to the intussusception.

**Fig. 3:**
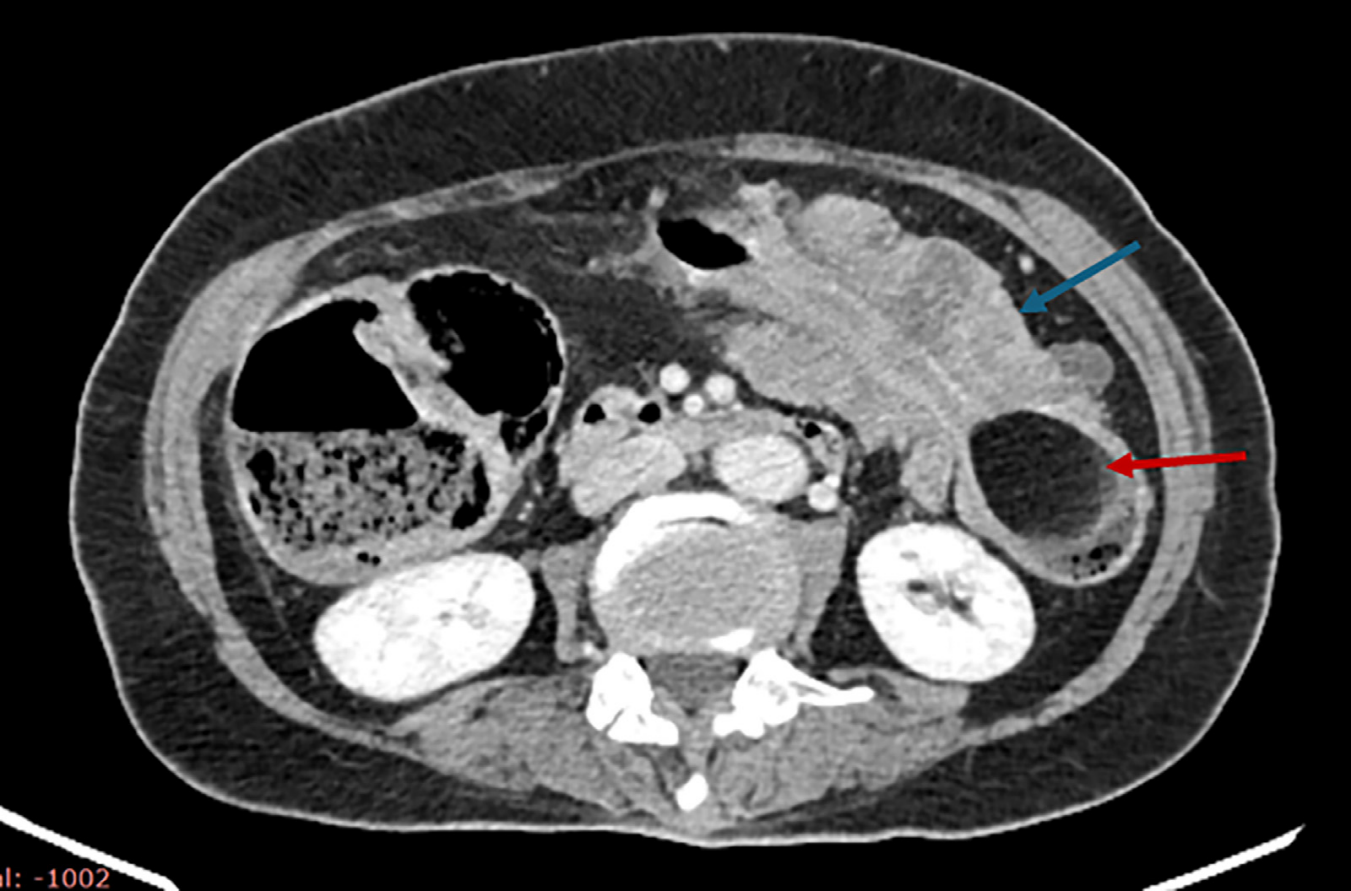
Axial contrast-enhanced CT showing colocolic intussusception (blue arrow). The previously identified fat-density lesion remains nonenhancing, further supporting its benign nature as a submucosal lipoma acting as the lead point (red arrow).

**Fig. 4:**
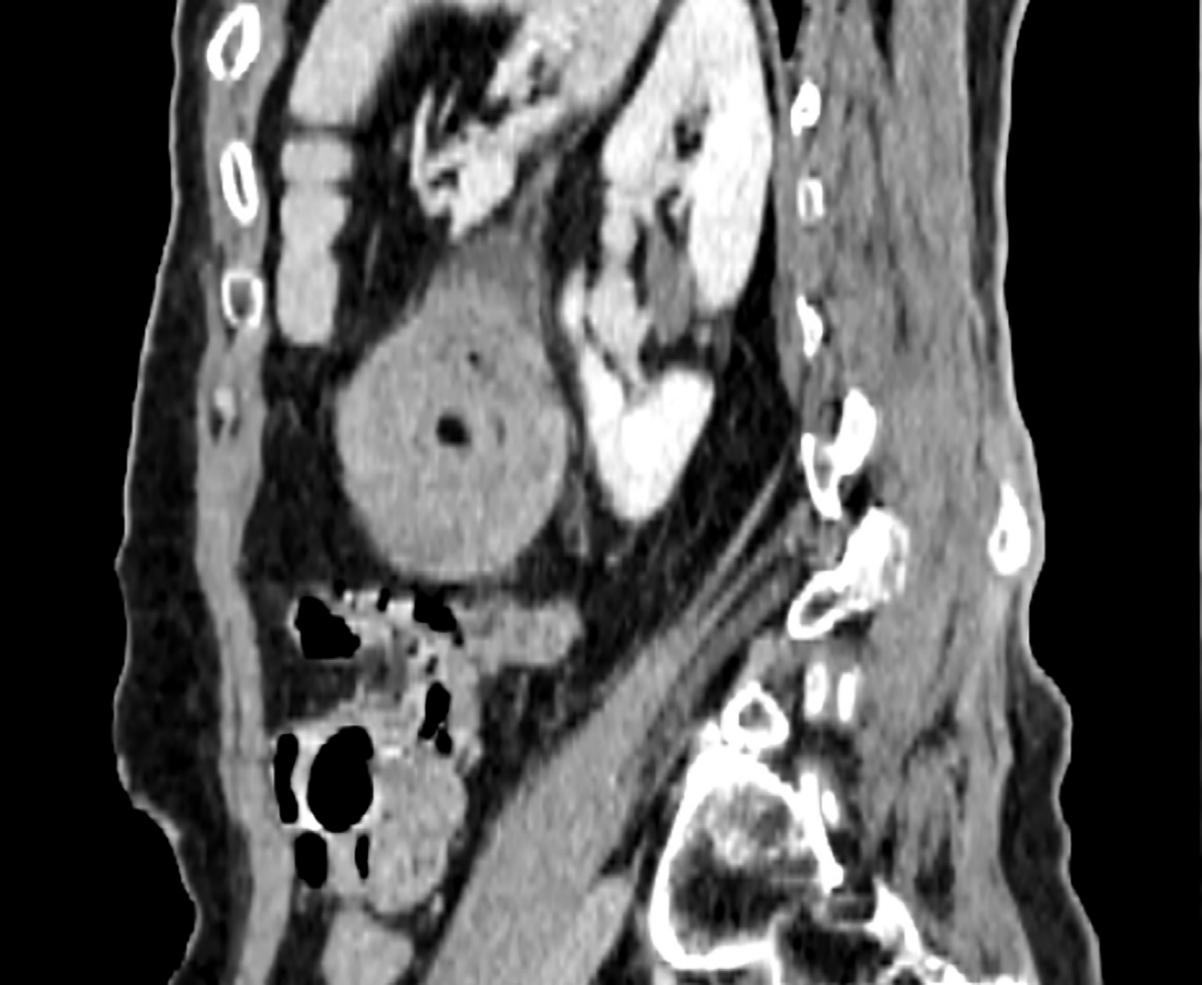
Sagittal oblique contrast-enhanced CT image showing a classic *target sign* (red arrow) corresponding to the intussusception. The concentric bowel wall layers and central mesenteric fat are clearly visualized, with the section aligned along the long axis of the invagination.

Initial imaging included an abdominal ultrasound, which revealed an acute left-sided colocolic intussusception with thickened bowel walls. A subsequent axial nonenhanced CT scan identified the lead point as a well-defined, intraluminal fat-density lesion consistent with a submucosal colonic lipoma (Fig. 1). Contrast-enhanced CT then demonstrated significant distension of the right and transverse colon, measuring up to 9 cm in diameter near the cecum (Fig. 2), as well as colocolic intussusception with the nonenhancing lipoma visible at its center (Fig. 3). A sagittal oblique CT reconstruction confirmed the diagnosis by showing the characteristic target sign with concentric bowel layers and central mesenteric fat (Fig. 4).

There were no signs of bowel ischemia or perforation, as evidenced by the absence of pneumoperitoneum or other complications such as free air. The patient was subsequently scheduled for surgical intervention. Surgical exploration did not find any sign of bowel ischemia. Then, surgical intervention included resection of the affected colonic segment and manual anastomosis. Postoperative recovery was uneventful, and the patient was stable without further complications.

Histological examination of the excised tissue revealed benign adipocytic proliferation within the colic wall.

## Discussion

Gastrointestinal lipomas are benign mesenchymal tumors, representing approximately 4% of benign GI tumors, with the colon being the most commonly affected site. Their incidence in the general population is estimated between 0.2% and 4.4% [5]. Colonic lipomas are most frequently found in individuals aged 50-70 years and show a slight female predominance. About 90% arise from the submucosa, while 10% originate from the subserosa. Most lipomas are asymptomatic and discovered incidentally; however, large or pedunculated lesions can lead to complications such as hemorrhage, obstruction, or intussusception [6]. Intussusception occurs in fewer than 5% of adults with colonic lipomas, most commonly when the lesion exceeds 4 cm and has a pedunculated morphology [7].

In adults, intussusception is typically caused by a structural lesion acting as a lead point. Colonic lipomas can function as such a lead point by mechanically interfering with peristalsis, leading to telescoping of the proximal bowel segment into the distal one [8].

This process involves the intussusceptum dragging along its mesentery into the intussuscipiens, potentially causing vascular compression, venous congestion, and bowel wall edema. Such mechanical distortion is particularly likely in lipomas with a pedunculated configuration, which creates a mobile, protruding mass susceptible to invagination during bowel movement. The invagination compromises vascular perfusion, potentially causing ischemia or full-thickness obstruction, which necessitates prompt intervention. Unlike pediatric cases, where intussusception is often idiopathic, in adults, more than 85%-90% of cases are associated with a pathological trigger, making identification of the etiology critical for guiding surgical management [9,10].

The clinical presentation of adult colonic intussusception due to lipoma is often subtle and nonspecific, contributing to diagnostic delays. Common symptoms reported in the literature include abdominal pain, nausea, vomiting, and changes in bowel transit, such as constipation or diarrhea. Some patients present with occult or overt gastrointestinal bleeding, while others may report nonspecific symptoms like bloating, fatigue, or weight loss in chronic cases [11,12]. In more advanced or acute settings, a full-blown obstructive syndrome may occur, characterized by cessation of bowel movements, abdominal distension, and colicky pain [8]. A key diagnostic challenge lies in the episodic nature of the symptoms. In cases of transient or partial intussusception, the lesion may reduce spontaneously, leading to fluctuating symptoms that mimic functional gastrointestinal disorders or partial obstruction from diverticular disease or malignancy. For this reason, maintaining a high index of suspicion is crucial in patients with recurrent or unexplained abdominal complaints [13]. In our case, the patient presented with a 6-day history of complete bowel obstruction and abdominal pain, a presentation consistent with the more advanced symptomatic spectrum described in the literature.

In this context, imaging plays a pivotal role in confirming the diagnosis and identifying the underlying cause. CT scanning remains the most sensitive and specific tool for diagnosing intussusception in adults. It enables clear anatomical visualization of the intussuscepted segment and can identify the typical “target” or “doughnut” sign, detect the lead point, and assess complications such as ischemia or perforation [14]. On axial sections, the intussusception typically appears as a concentric “target” sign, while sagittal or coronal views may show a “sausage-shaped” configuration corresponding to the telescoped bowel [15]. In our case, the characteristic fat attenuation of the lead mass on CT confirmed a lipoma, with values between –40 and –120 Hounsfield units. The absence of surrounding fat stranding, wall thickening, or lymphadenopathy made a benign diagnosis likely, but resection remained indicated due to the intussusception and potential risk of malignancy [8].

Other imaging modalities can also provide useful insights. Ultrasound may reveal a “target” or “pseudo-kidney” sign, particularly in early evaluation, though its role is more established in pediatrics [16]. However, in experienced hands, abdominal ultrasound can occasionally detect adult intussusception, especially in settings where CT is unavailable or contraindicated, offering a rapid, radiation-free alternative. MRI offers high-resolution contrast for soft tissue evaluation, but is rarely used in emergency settings. CT colonography can identify the lesion’s fat density, morphology, and location with high accuracy [17]. Colonoscopy remains valuable in the diagnosis of submucosal lesions and, in select cases, permits endoscopic removal of small pedunculated lipomas [18]. In addition to diagnosis, cross-sectional imaging plays a critical role in preoperative planning by delineating lesion size, location, and associated complications, allowing the surgical team to anticipate the extent of resection and choose the most appropriate operative approach.

Beyond diagnosis, a key challenge is distinguishing between benign and malignant lead points. While lipomas are generally benign, up to 75% of adult colonic intussusceptions are linked to malignancy, most commonly adenocarcinoma [9,19]. Even when imaging favors a benign lesion, liposarcoma cannot always be excluded, especially in large or atypical masses [20]. Therefore, surgical resection without prior reduction is often recommended to minimize oncological risk and ensure definitive treatment [21].

These considerations directly influence therapeutic decisions, making surgical resection the gold standard for treating adult intussusception, particularly when the colon is involved. The objectives of surgery are to relieve bowel obstruction, eliminate the lead point, obtain a definitive histopathological diagnosis, and prevent recurrence or complications. Depending on the lesion’s location and intraoperative findings, surgical options include segmental colectomy, right or left hemicolectomy, and either laparoscopic or open approaches. In colonic intussusception, preoperative reduction is generally discouraged because of the potential risk of tumor dissemination. However, in selected cases with a presumed benign etiology and favorable anatomy, laparoscopic-assisted resection has been shown to be both feasible and safe, offering the advantages of faster recovery and reduced morbidity [22,23]. In our case, segmental colectomy was performed without prior reduction, consistent with the current literature, which emphasizes immediate surgical intervention even when a benign lesion such as a lipoma is suspected, due to persistent diagnostic uncertainty and the potential for rapid clinical deterioration [21]. Minimally invasive techniques are increasingly adopted and are particularly suitable for hemodynamically stable patients with well-localized, benign-appearing lesions, provided adequate imaging and surgical expertise are available.

The prognosis following surgical resection of a colonic lipoma causing intussusception is generally excellent. In most documented cases, including ours, patients recover fully with no recurrence [24]. Favorable outcomes are strongly associated with early diagnosis, absence of bowel ischemia or perforation, complete surgical resection, and benign histopathology.

Routine postoperative surveillance imaging is not typically indicated when the histological findings confirm a benign lipoma. However, patients with persistent gastrointestinal symptoms, significant comorbidities such as inflammatory bowel disease, or a family history of gastrointestinal malignancies may benefit from individualized follow-up strategies [20]. In our case, the patient had no residual symptoms postoperatively, and no recurrence was observed. Her management followed the best practices outlined in recent literature, avoiding unnecessary delay and ensuring a definitive resolution.

## Conclusion

Adult colocolic intussusception is a rare but clinically significant condition that often results from a pathological lead point. In our case, a submucosal colonic lipoma measuring over 4 cm acted as the trigger, leading to acute bowel obstruction. CT imaging played a pivotal role in identifying both the intussusception and its benign fatty origin, guiding prompt surgical decision-making. Given the risk of malignancy and the limitations of imaging alone, surgical resection remains the treatment of choice. Early recognition and timely intervention are essential for favorable outcomes, especially in elderly patients presenting with nonspecific, recurrent abdominal symptoms.

## Patient consent

Written informed consent for the publication of this case report was obtained from the patient.
